# Adrenal Crisis Secondary to Influenza and Tetanus Vaccination in an Adult Without Known Adrenal Insufficiency: A Case of Autoimmune Adrenalitis

**DOI:** 10.7759/cureus.16312

**Published:** 2021-07-11

**Authors:** Shiwani Kamath, Jessica K Khabra, Parth Desai, Johnathan Frunzi

**Affiliations:** 1 Internal Medicine, Medical Center of Trinity, Trinity, USA; 2 Critical Care Medicine, Medical Center of Trinity, Trinity, USA

**Keywords:** adrenal crisis, primary adrenal insufficiency, post-vaccination, autoimmune adrenalitis, seasonal influenza vaccine, tetanus vaccine

## Abstract

Adrenal crisis (AC) is a rare but known life-threatening condition in patients with adrenal insufficiency (AI). We report the case of a 21-year-old without known AI who developed AC after routine vaccinations. Workup revealed that the patient had underlying, undiagnosed autoimmune adrenalitis. This is the first report of AC induced by influenza and diphtheria, tetanus, and acellular pertussis (DTaP) vaccinations in a patient without known AI.

## Introduction

Acute adrenal insufficiency (AI), also known as adrenal crisis (AC), is an emergency characterized by a sudden lack of cortisol production [1]. AC is an acute complication of chronic AI, which may remain undiagnosed until overwhelming factors result in a crisis state [2]. In a study by Hahner et al., the incidence of AC in patients with known AI was found to be 8.3 per 100 patient-years, with mortality related to AC of 0.5 per 100 patient‐years for patients with AI [3]. Major precipitating factors of AC include infections, trauma, surgery, and emotional stress [1]. Those with other glandular endocrinopathies are at a higher risk of developing AI [1]. Notable manifestations of AC are fatigue, weight loss, reduced appetite, nausea, vomiting, and abdominal pain. On physical examination, patients may have hypotension or shock that is refractory to fluid challenges and vasopressors, tachycardia, and skin hyperpigmentation [1]. Remarkable initial laboratory findings include hyponatremia, hyperkalemia, recurrent hypoglycemia, and elevated creatinine levels, possibly secondary to acute kidney injury [1]. Further investigations, depending on the etiology of the AI, may demonstrate high or normal adrenocorticotropic hormone (ACTH), low aldosterone, or high renin [1]. Definitive treatment of AI involves steroids with aggressive fluid and vasopressor administration in the intensive care unit (ICU) setting [1]. Although hydrocortisone is the steroid of choice, dexamethasone, prednisolone, or methylprednisolone may also be used [1]. Until the cause is elicited, the patient should receive coverage with empiric antibiotics [1]. It should be noted that all laboratory studies should be conducted prior to the administration of steroids [1]. In this report, we present a case of AC triggered by influenza and tetanus immunization in an adult without prior history of AI.

## Case presentation

The patient was a 21-year-old Caucasian male who presented to the emergency room (ER) with complaints of generalized body aches, fatigue, and malaise for five days. He reported mid-abdominal discomfort associated with scant vomiting for two days with associated decreased appetite and fluid intake. He endorsed tingling and numbness of feet and toes for one day; his family had brought him to the hospital as he had been lethargic and appeared cyanotic. He denied fever, chills, rash, joint swelling, joint pain, neck stiffness or pain, sore throat, sinus drainage or pressure, urinary complaints, or bowel changes. He also denied recent travel or sick contacts. The patient had been vaccinated with influenza and diphtheria, tetanus, and acellular pertussis (DTaP) vaccines one week ago in preparation for school. In the ER, the patient was lethargic, bradycardic with a heart rate of 30 beats per minute, and had low blood pressure at 90/60 mmHg. On examination, he had a cold, as well as clammy skin that was mottled in color. Initial laboratory tests are presented in Table 1.

**Table 1:** Initial laboratory testing at the time of admission AST: aspartate aminotransferase; ALT: alanine aminotransferase

Laboratory test	Result	Normal range
Hemoglobin (g/dL)	16.9	13.7-17.5
Sodium (mmol/L)	102	136-145
Potassium (mmol/L)	6.2	3.5-5.1
AST (U/L)	299	15-37
ALT (U/L)	124	30-65

Additionally, his urine analysis was unremarkable. He was transferred to the ICU and started on fluids and given a prophylactic dose of ceftriaxone. A cortisol level was drawn, and the patient was given a dose of dexamethasone for the possibility of AC in light of concurrent hyponatremia, hyperkalemia, hypotension, and hypothermia. His blood pressure, heart rate, and coloration improved. Due to this positive response, he was started on intravenous (IV) hydrocortisone. Subsequent laboratory tests are summarized in Table 2.

**Table 2:** Subsequent laboratory testing ACTH: adrenocorticotropic hormone; DHEA: dehydroepiandrosterone sulfate; TSH: thyroid-stimulating hormone

Laboratory test	Result	Normal range
Cortisol (mcg/dL)	0.69	4.3-22.4
ACTH (pg/mL)	>2000	7.2-63.3
DHEA (mcg/dL)	<15	80.0-560.0
Total testosterone (ng/dL)	200	264-916
Free testosterone (pg/mL)	0.8	9.3-26.5
21-hydroxylase (U/mL)	40	<1
TSH (mcInU/mL)	5.25	0.36-3.74
T4 (ng/dL)	1.62	0.89-1.76

The low cortisol level prior to the treatment combined with improvement in clinical status following glucocorticoid treatment was consistent with AC. Further diagnostic laboratory testing demonstrated an elevated ACTH along with a decreased dehydroepiandrosterone sulfate (DHEA), total testosterone, and free testosterone. 21-hydroxylase antibodies were elevated, consistent with autoimmune Addison’s disease. Thyroid-stimulating hormone (TSH) was slightly elevated, with a normal T4, suggestive of euthyroid sick syndrome. CT abdomen done showed no gross adrenal enlargement or abnormalities. Blood cultures did not grow any organisms. With continued treatment with steroids, the patient’s severe hyponatremia and other electrolyte abnormalities were corrected. The endocrinologist recommended starting the patient on maintenance mineralocorticoid on discharge.

## Discussion

Our patient did not have a known history of AI prior to admission, nor did he endorse any symptoms prior to his vaccination. However, we speculate that our patient had underlying undiagnosed AI, as a study by Bleicken et al. demonstrates that 20% of symptomatic patients may remain undiagnosed for over six months [4]. This may be attributed to the already vague symptoms of AI, such as fatigue, weight loss, and loss of appetite, which develop slowly over months [4]. In itself, AI is fairly rare with studies reporting the prevalence ranging from 35-60 per million [5-7], thereby making the diagnosis easier to be overlooked. Patients with AI may go undiagnosed until they have significant stressors inducing an AC [4]. Per Ghada et al., AC is always a complication of AI, and the underlying etiology must be determined [1]. In 70-90% of cases of AI, the primary etiology was determined to be autoimmune adrenalitis [5]. As our patient had antibodies against 21-hydroxylase, we believe he had underlying autoimmune Addison’s disease. It is important during the time of diagnosis to also assess for other endocrine disorders, as they can occur concurrently and further precipitate AC [1]. Commonly associated disorders include thyroid disease, diabetes mellitus, and, in women, premature ovarian insufficiency [5].

We believe our case is the first presentation of AC induced by influenza and DTaP vaccination in a patient without a known history of AI. Potential causes of AC are usually well-known, with ongoing debates of less common etiologies and arguments around what constitutes a significant stressor. Some data suggests that even strong emotional states can precipitate AC [8]. One case study has described a situation similar to ours, and it involves a patient who developed AC after receiving DTaP, with the notable difference being that the patient had known pre-existing Addison’s disease [9]. We also consider that our patient had received two vaccines simultaneously, potentially increasing the stressor burden.

Typically, for patients with a known history of AI who present with fatigue and gastrointestinal disturbances along with hypotension, AC should be high on the differential [1]. All vaccines are known to cause fever, fatigue, and gastrointestinal disturbances by themselves, thereby making it difficult to diagnose AC [9]. In patients such as ours without a known history of AI who present with shock resistant to vasopressors, AC should be ruled out [1]. Regardless of a clear diagnosis, empiric treatment with steroids should take priority over confirmatory testing in cases of suspected AC [1].

As our patient did not have any prior history, he was educated on the course of the disease and the need for exogenous steroids for maintaining good health [1]. As infections are a major precipitant of AC, receiving future routine vaccinations should not be barred in patients with AI or those who have AC secondary to vaccinations [9]. Rather, Major et al. have suggested that there may be a role for stress-dose steroids prior to vaccinations in these patients [9].

## Conclusions

This case demonstrates that vaccines can play the role of a stressor, potentially significant enough to induce AC. We recommend future studies to assess the incidence of AC in patients with and without known AI after vaccinations. We also believe that further studies on preventing AC after vaccinations and the role of stress-dose steroids in preventing AC as well as the efficacy of the vaccines are required.
